# Suffering-based medicine: practicing scientific medicine with a humanistic approach

**DOI:** 10.1007/s11019-019-09920-8

**Published:** 2019-08-20

**Authors:** Auro del Giglio

**Affiliations:** Faculdade de Medicina da Fundaçao ABC, Rua Príncipe de Gales 821, Santo André, 09060-870 Brazil

**Keywords:** Medical philosophy, Physician–patient relation, Humanism, Humanities

## Abstract

Suffering, defined as a state of undergoing pain, distress or hardship, is a multidimensional concept; it can entail physical, psychological and spiritual distress that prompts the sufferer to seek medical attention. As a construct originating from and unique to each patient, no patient’s suffering is equal to another’s or completely reducible to any generalizable frame of understanding. As it happens in a common medical encounter, the suffering patient requires an anamnesis provided by attentive and comprehensive listening to both the said and unsaid parts of his or her discourse interpreted through the hermeneutical skills of the physician. Suffering can then be decoded into a complex construct, which can guide the formulation of a strategy to help patient coping by attempting to find meaning in it. To help this search for meaning, one may employ philosophic therapy, bibliotherapy and counseling, among other approaches. Suffering-based medicine (SBM) thus circumvents the limitations of the reductionistic Allopathic medical frame of understanding of disease. Such an attitude should be a common goal for all scientific medicine practitioners, as it complements and does not conflict with any of its therapeutic modalities. Furthermore, medical education should include humanistic disciplines to empower physicians to better understand their patients’ experience of suffering.

## Introduction

The Oxford dictionary defines suffering as a state of undergoing pain, distress or hardship (Oxford Dictionaries 2018b). Even though most commonly associated with pain, suffering involves various other dimensions in addition to the physical, including spiritual, psychological, familiar, social and cultural factors (Cassel 1982).

Scientific medicine, amid its recent exponential development, presents the prospect of an all-in-one tool to cure disease. However, a multidimensional concept such as suffering—which almost always accompanies disease—cannot be completely encompassed by a reductionist view focused solely on the evaluation and treatment of physical complaints. As a medical oncologist, teaching students, residents and fellows, as well as directly caring for patients for the last 30 years, it is clear to me that scientific medicine needs to expand its scope if we are to improve quality of care and increase patient satisfaction.

Unfortunately, despite recent scientific advances, there are still dimensions of the patient qua person that are not addressable by a mechanistic (Wulff et al. 1990) model of disease causation, typical of current medical practice. However, if in addition to understanding physical disease on a scientific basis, we also try to focus on the suffering of the patient, a much more comprehensive paradigm opens itself to study, which we will call suffering-based medicine (SBM). Through SBM, an expanded view of the patient’s disease and the suffering that accompanies it, we can diagnose and treat the disease as we usually do in the context of scientific medicine while also addressing all other dimensions of the suffering experienced by the patient. Furthermore, when we extend our view to include suffering, we also obtain clues to the usefulness of additional therapeutic strategies not pertinent to traditional scientific medicine, such as counselling, bibliotherapy and philosophical therapy.

Interestingly, SBM does not interfere at all with the practice of scientific medicine, nor with the various complementary/alternative medicine approaches. This lack of interference depends on a basic difference of focus of SBM. While other, oftentimes conflicting, types of medical practices (alternative/complementary and traditional scientific) focus on diverse ways of understanding disease, SBM concentrates on the suffering experienced by the patient. Therefore, SBM, in addition to not interfering with these widely distinct types of practices, can also enhance them.

In this article, we will initially analyze suffering phenomenologically to better understand the object of SBM. We will try to define SBM and then attempt to compare it with several other current paradigms in vogue, such as evidence-based medicine, person-based Medicine, and narrative medicine. Then, when considering how a medical encounter typically unfolds, we will reflect on the practical application of SBM, starting with the anamnesis of the suffering patient, followed by an attempt at understanding the suffering experienced by the patient. Finally, we will address how we can envision additional therapeutic strategies for this patient based on the understanding of his or her suffering.

## Phenomenology of suffering associated with illness

Phenomenology is a discipline of philosophy focused on the study of the structures of consciousness as experienced by the individual. Therefore, phenomenology is essentially subjective, claiming to describe on the first-person level several types of experiences (phenomena). Categories of experience addressed by phenomenology include memory, thought, perception, desire, volition, bodily awareness and emotion. Implicit to this subjective descriptive act is the intentionality of the one who experiences, as all experiences are necessarily experiences of something. Intentionality, in turn, projects consciousness toward phenomena through thoughts, concepts, and images, thus imparting meaning to experiences (Smith 2016).

Essential to the phenomenological method is reduction, also understood as a form of distancing oneself from the phenomena under examination. As phenomenology aims at a direct description of our experience, we have to somehow distance ourselves from (like putting within brackets) “everyday given understanding…leaving behind our general modes of interpretation and conventional meaning-generating practices in order to genuinely examine a given phenomenon. We thus bracket not only the conventional and social meanings of a phenomenon, but also the personal meanings one has been accustomed to attach to phenomena, in order to re-examine them” (Carel 2012).

Since phenomenology allows for the description of the embodied experience of illness, it can help us to understand how illness alienates the sick person from his or her usual world through the limitations it imposes and the consequent suffering it engenders. Furthermore, phenomenology also allows us to analyze the meaning the ill person ascribes to all these new life experiences brought about by illness. Despite being subjective and therefore peculiar to each and every ill person, the experience of illness has some common characteristics including the perception of loss of wholeness, loss of certainty and control, loss of freedom to act, and loss of the familiar world (Toombs 1987).

In the context of illness, suffering arises as a combination of unpleasant physical sensations such as pain, shortness of breath, and nausea, as well as from failure to achieve predefined life goals due to a break in the life narrative that was in development before the patient became ill. Suffering produces a narrowing of the ill person’s existential amplitude, of his or her being in the world. As described by Svenaeus (2014): “In physical suffering, the world is typically narrowed down: I am forced to focus my attention on the body that hurts and have problems focusing on other things. However, this does not mean that the pain experience would concern only the body. The structure of the world around us changes in physical suffering: the person who suffers perceives things around her in new ways”. In fact, Svenaeus (2014) understands suffering brought about by physical illness as an alienating mood that can potentially modify the entire way one feels in the world with respect to one’s embodiment, relationships to others and to one’s own values. Therefore, because of the narrowing of the ill person’s existential amplitude (“being in the world”) either due to physical discomfort or other limitations, suffering distorts the way in which the person sees himself and how he is seen by others, making his life narrative up to this moment potentially incoherent with his life from now on.

In sum, suffering poses new existential challenges to the sufferer that may substantially alter his or her way of looking at and being seen by the people surrounding him or her. This new way of relating to the world and to his or her acquaintances is imbued, in turn, by the sufferer’s cultural background, religious beliefs, socioeconomic constraints, and so on (Svenaeus 2014).

A construct may be defined as “an idea or theory containing various conceptual elements, typically considered to be subjective and not based on empirical evidence” (Oxford Dictionaries 2018a). We propose that suffering can be understood as a construct that must be related from the sufferer to the healer through a detailed anamnesis. It has then to be further understood and interpreted by the healer for him or her to be able to indicate to the sufferer new ways of coping.

The patient suffering construct needs to be interpreted by the physician to be understood. With the help of hermeneutics—defined as “the study of the methodological principles of interpretation” (Merriam-Webster 2018)—one can start attempting to interpret the suffering construct offered by the patient during anamnesis. Such an understanding may open up ways to decrease suffering by changing a person’s values and goals to reinterpret/rebuild one’s life history, the continuity of which was broken by the advent of illness (Svenaeus 2014). It allows for a redemptive, so to speak, capability of suffering, with its discomforts and limitations, through the reshaping of life goals and redesigning of one’s narrative to find meaning again in this new and different world. Furthermore, listening to the cry of the sufferer awakens an ethical obligation in others to attend to the sufferer, as beautifully indicated by Levinas (1988). Nevertheless, according to this author, this ethical obligation to care for the sufferer justifies our existence as doctors (Levinas 1988).

What becomes clear is that modern clinical medicine, within its mechanical model of understanding disease (Wulff et al. 1990), has no tools to deal with the many nonphysical dimensions of suffering. If one wants a fuller understanding of suffering experienced by patients, he or she may need then to bring to the fore other humanistic sciences such as sociology, psychology, anthropology, history, philosophy and theology (Bueno-Gómez 2017). This type of humanistic knowledge is important for a fuller hermeneutic understanding of the patient’s suffering construct presented to the physician during anamnesis. Furthermore, the need to have basic principles of these humanistic disciplines may lead to a remodeling of the medical school curriculum.

In the following section, I will try to show how understanding a patient’s suffering construct in the context of SBM can offer a humanistic approach to the patient while circumventing some of the insufficiencies of the scientific medical approach to dealing with disease and illness.

## How we practice SBM

As we saw earlier, allowing a suffering construct to be formulated and offered by the patient through a more comprehensive anamnesis occurs in parallel to the collection of clinically relevant data needed for both a diagnosis and a therapeutic plan within the frame of scientific medicine. In addition to the longer time required for both tasks, there is no interference between them, as we see the suffering construct being conveyed by the patient as a completely and conceptually independent activity from the procuring of clinical data by the physician. Furthermore, especially when faced with chronic disease, the information regarding the suffering that the patient is experiencing may be collected progressively throughout the multiple visits that will follow the initial one.

The second step of SBM is the hermeneutic understanding of the suffering construct, which is also parallel to and completely independent from the diagnostic step of scientific medicine. Here, we try to interpret the patient’s suffering construct with the aim of understanding it in the context of his or her cultural, psychological, anthropological, sociological and spiritual peculiarities. Therefore, some knowledge of these humanistic disciplines is necessary to enable the physician to interpret whatever is conveyed by the patient as his or her suffering construct.

The final step of SBM is the formulation of potentially useful therapeutic adjunct measures based on the hermeneutical understanding of the suffering construct exposed by the patient. Despite the interventional nature of this step, which resembles the therapies offered by scientific medicine, it is conceptually completely independent of those therapies. When we envision therapeutic adjunct measures in the context of SBM, we think broadly of the capacity of the physician to advise patients on their life difficulties experienced in the context of their illnesses. Helping another suffering subject to proactively find meaning in his or her distressful illness experience can be achieved through attentive listening and counseling (Gillies and Neimeyer). Furthermore, this advising role of the physician may also involve expanded therapeutic possibilities exploring the potential healing effects of reading (bibliotherapy), reflection (philosophic therapy), music (musicotherapy), art (art therapy), and related approaches. All of these potential therapeutic adjunct measures predicated on the hermeneutical understanding of the suffering construct offered by the patient have no theoretical or practical interference whatsoever with the conventional therapies of scientific medicine.

## How does SBM compare to other medical paradigms

SBM differs from evidence-based medicine (EBM) (Sackett and Rosenberg 1995), defined as “the conscientious explicit and judicious use of current best evidence in helping individual patients make decisions about their care in the light of their personal values and beliefs”. While EBM recognizes the fundamental role of patients’ personal values and beliefs in making decisions, it is focused on finding adequate evidence to solve clinical problems. SBM, in turn, focuses on all nonphysical personal aspects of the illness experience not amenable to being solved by finding the best evidence. Even though EBM can and must be the base of scientific medicine practice, it cannot account for all of the other nonorganic aspects of the illness experience that concern patients and physicians.

SBM also differs from person-centered medicine (Braš et al. 2011), defined as “a humanistic, biopsychosocial perspective, combining ethical values on ‘the ideal physician’, with psychotherapeutic theories on facilitating patients’ disclosure of real worries, and negotiation theories on decision making.” Person-centered medicine “puts a strong focus on patient participation in clinical decision making by taking into account the patients’ perspective, and tuning medical care to the patients’ needs and preferences”. SBM does not seek to structure patients’ views of their suffering using any specific framework but rather to have the patient convey his or her suffering construct in whatever frame of understanding he or she may have of his or her illness experiences. Thus, despite the possibility of being understood differently by diverse physicians, the suffering construct is elaborated by the patient, and it is from its hermeneutical interpretation by the physician that the patient’s values, vision and meaning all emerge.

Likewise, we can say that SBM is different from narrative medicine (Zaharias 2018) to the extent that the suffering construct of a patient may involve a narrative but is much more complex than a history abstracted from the patient’s anamnesis. One may interpret from the construct without necessarily using an orderly and successive coordination of facts. Furthermore, the necessity of clarity characteristic of a coherent narrative sometimes presents itself as an artificial arrangement of facts and events by the physician based on scattered information collected from the patient’s anamnesis.

We thus have in SBM not a new way of practicing scientific medicine but a philosophically consistent and truly complementary approach to account for the nonphysical necessities of the suffering subject. Homeopathic, Ayurvedic and Chinese medicines, for example, have ways of understanding disease that are completely diverse and philosophically inconsistent with scientific medicine. Therefore, it is impossible for the same physician simultaneously to practice scientific medicine and any of these alternative/complementary approaches. The same applies to any other alternative/complementary approach that has different ways of conceptually understanding disease that are not compatible with scientific medicine. There is no conflict, however, between SBM and scientific medicine due to their focus on different objects (suffering construct and disease, respectively) and to the distinct theoretical background that each one uses. SBM focuses on the suffering construct offered by the patient and uses several humanistic disciplines to hermeneutically understand it, while scientific medicine focuses on the physical aspects of the disease that are explainable in the context of a physio-pathological, scientifically oriented method.

SBM is not intended as a substitute for scientific medicine. In fact, it presupposes the practice of scientific medicine with its diagnosis and treatment, both deemed essential for the patient to improve clinically. SBM expands the scope of scientific medicine, however, to better address the nonphysical symptoms that constitute the suffering construct offered by the patient. SBM will only be effective if fully integrated to scientific medicine.

SBM is not new conceptually. In the history of medicine, the more impotent our colleagues’ therapeutic armamentarium, the more they employed techniques such as advising patients and maybe attended to the nonphysical aspects related to their suffering. We read in the literature, for example, several examples of medical advice, including suggestions for patients to entertain trips to different places, changes of residence, etc., as adjunct curative approaches. In addition, counseling by physicians or priests and reading, writing and reflecting as therapeutic additions are as old as the practice of medicine. The difference, from my point of view, is that SBM needs explicit recognition as a valid, structured and reproducible complement to scientific medicine and needs its practice stimulated alongside it. Furthermore, we need to include in the medical curriculum for young physicians humanistic disciplines that may help them to become better interpreters of their patients’ suffering constructs, in addition to their first concern of treating symptoms arising from clinical disease.

## Limitations of SBM

SBM presupposes the possibility of obtaining through anamnesis a suffering construct from the patient. Therefore, SBM cannot be practiced in full with children unable to verbalize their feelings, or with noncooperative or intellectually deficient adults unable to entertain a dialogue with the physician. Aside from these limitations that pertain to the doctor–patient relationship, SBM can be practiced outside these borders, aiming at caregivers and relatives of these patients.

SBM is a modality that is much more appropriate for chronic debilitating diseases because suffering, albeit present in cases of acute disease, may be short-lasting, never becoming a construct. Chronic disease, when asymptomatic and nonlimiting to patients’ activities, may also fail to produce a structured form of suffering that can be addressed by SBM. We see, therefore, that SBM should be entertained for select patients who can benefit from its application.

SBM should not interfere with the current practice of non-medical professionals dedicated to the psychosocial-spiritual care of patients such as psychologists, chaplains, social workers, etc. SBM is solely an effort to expand the physician’s approach to the non-organic aspects of medical care.

## Conclusions

SBM allows the physician to capture through anamnesis and structure through hermeneutics a suffering construct for each patient, helping him to find meaning in his illness experience and thus improve his coping skills. It is a complementary and nonconflicting praxis to be incorporated into scientific medical practice. SBM stands to improve both patient and physician satisfaction, enriching medical encounters with humanistic considerations. However, for physicians to deal adequality with these considerations, changes in medical education may be required.
